# Annotation of the Asian Citrus Psyllid Genome Reveals a Reduced Innate Immune System

**DOI:** 10.3389/fphys.2016.00570

**Published:** 2016-11-29

**Authors:** Alex P. Arp, Wayne B. Hunter, Kirsten S. Pelz-Stelinski

**Affiliations:** ^1^Citrus Research and Education Center, Department of Entomology and Nematology, University of FloridaFort Pierce, FL, USA; ^2^U.S. Horticultural Research Lab, Agricultural Research Service, United State Department of AgricultureFort Pierce, FL, USA

**Keywords:** *Diaphorina citri*, immune, genes, Imd, liberibacter, antimicrobial peptide, virus

## Abstract

Citrus production worldwide is currently facing significant losses due to citrus greening disease, also known as Huanglongbing. The citrus greening bacteria, *Candidatus* Liberibacter asiaticus (*C*Las), is a persistent propagative pathogen transmitted by the Asian citrus psyllid, *Diaphorina citri* Kuwayama (Hemiptera: Liviidae). Hemipterans characterized to date lack a number of insect immune genes, including those associated with the Imd pathway targeting Gram-negative bacteria. The *D. citri* draft genome was used to characterize the immune defense genes present in *D. citri*. Predicted mRNAs identified by screening the published *D. citri* annotated draft genome were manually searched using a custom database of immune genes from previously annotated insect genomes. Toll and JAK/STAT pathways, general defense genes Dual oxidase, Nitric oxide synthase, prophenoloxidase, and cellular immune defense genes were present in *D. citri*. In contrast, *D. citri* lacked genes for the Imd pathway, most antimicrobial peptides, 1,3-β-glucan recognition proteins (GNBPs), and complete peptidoglycan recognition proteins. These data suggest that *D. citri* has a reduced immune capability similar to that observed in *A. pisum, P. humanus*, and *R. prolixus*. The absence of immune system genes from the *D. citri* genome may facilitate *C*Las infections, and is possibly compensated for by their relationship with their microbial endosymbionts.

## Introduction

Insects transmit a wide range of animal and plant pathogens. The majority of vector-borne pathogen research has focused on viral zoonotic and plant pathogens, with commensurate attention given to understanding the physiological factors that promote interactions between the pathogen and vector (Fereres and Moreno, 2009; Sim et al., 2014). Insects lack the adaptive immune system found in chordates, and instead rely exclusively on an innate immune system to regulate interactions with invading microorganisms. The insect innate immune system consists of humoral and cellular defense responses activated by pattern recognition receptors (PRRs), which detect and bind to conserved microbial surface structures, called pathogen-associated molecular patterns (PAMPs), (Pili-Floury et al., 2004; Dziarski and Gupta, 2006; Wang et al., 2011).

Holometabolous insects, including *Drosophila, Anopheles* spp. (Diptera), and *Tribolium castaneum*, (Coleoptera) have served as important models for genomic and functional investigations of insect innate immunity (Christophides et al., 2002; Hoffmann and Reichhart, 2002; Roth et al., 2010). The high level of similarity in immune function shared among these insects, other non-insect arthropods, and vertebrates, is suggestive of a highly conserved innate immune system (Kang et al., 1998; McTaggart et al., 2009; Smith and Pal, 2014). In contrast, three hemimetabolous insects, the pea aphid, *Acyrthosiphon pisum* (Family: Aphididae), kissing bug, *Rhodnius prolixus*, (Family: Reduviidae) and human louse, *Pediculus humanus*, (Family: Pediculidae) possess comparatively reduced innate immune systems that lack genes for recognition and killing of Gram-negative bacteria (Gerardo et al., 2010; Kim et al., 2011; Mesquita et al., 2015).

Like *A. pisum*, the hemimetabolous Asian citrus psyllid, *Diaphorina citri* Kuwayama, (Hemiptera: Liviidae) is also host to a number of endosymbionts and the plant bacterial pathogen associated with citrus greening disease, *Candidatus* Liberibacter asiaticus (*C*Las) (Aubert, 1987; Thao et al., 2000; Halbert and Manjunath, 2004). Currently, citrus greening disease, or Huanglongbing, threatens worldwide citrus production (Hall et al., 2013). Psyllids transmit *C*Las, a Gram-negative alpha-proteobacteria, beginning a few days to a week after acquisition and for the lifetime of the vector (Pelz-Stelinski et al., 2010; Ammar et al., 2011). *D. citri* harbors *C*Las in a persistent-propagative manner, suggesting that *C*Las has developed mechanisms to avoid psyllid cellular and humoral immune defenses.

Circulative, persistent pathogens must traverse immunological and physical barriers in the midgut and salivary glands before they can be transmitted to plants during subsequent feeding. Within the midgut, the invading pathogen must pass through the peritrophic matrix (if present), survive exposure to antimicrobial peptides (AMPs) and reactive oxygen species (ROS), compete with resident gut bacteria, and pass though the gut epithelium (Ha et al., 2005; Charroux and Royet, 2010; Kuraishi et al., 2011). Once in the hemocoel, pathogens must avoid phagocytosis, encapsulation, melanization, and effectors produced by the fat body (Ratcliffe and Gagen, 1976; Lavine and Strand, 2002; Hillyer et al., 2003). To overcome these immune barriers, insect pathogens have evolved mechanisms to avoid the insect defenses, such as not expressing PAMPs, degrading effectors, inhibiting immune signaling pathways, or killing hemocytes (Apidianakis et al., 2005; Vallet-Gely et al., 2008). Understanding vector immune responses may lead to novel management strategies to reduce insect-transmitted pathogens (Reese et al., 2014). This is evident in the recently successful manipulation of mosquito immune responses through the introduction of *Wolbachia*, resulting in the upregulation of immunity genes and inhibition of dengue virus replication (Moreira et al., 2009; Pan et al., 2012).

**Figure 1:**
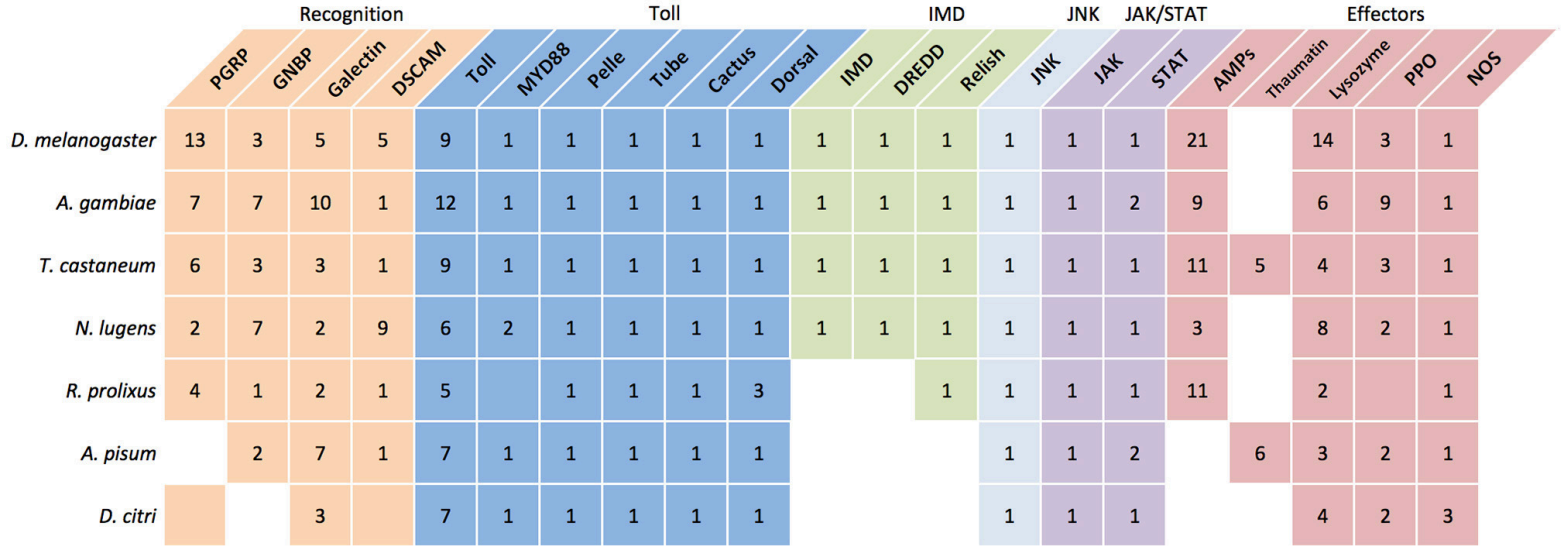
**A comparison of select immune associates genes in select model insects and *D. citri* suggests *A. pisum* and *D. citri* have similar immune pathway characteristics**. The number in each box represents the reported copy number of each gene. Empty boxes represent genes that have not been identified. Boxes that are colored but lacking a number are assumed to be present but the specific number is unknown or the potential gene function is putative. (Christophides et al., 2002; Sackton et al., 2007; Gerardo et al., 2010; Bao et al., 2013; Mesquita et al., 2015).

Mechanisms that facilitate *C*Las evasion of psyllid cellular and humoral innate immune defenses are unknown. We used the unpublished *D. citri* genome (Hunter et al., 2014) to annotate immune system genes and determine phylogenetic relationships with homologous sequences from other insects. Immune genes were automatically annotated with the NCBI Eukaryotic Automated Annotation Pipeline and manually annotated using a tblastx search of the predicted *D. citri* transcripts against a database of insect immune genes. Here, we illustrate that *D. citri* may resemble their close Sternorrhynchan relative, *A. pisum*, which have a reduced immune response toward Gram-negative bacteria compared to well-characterized homopterous insect models (Figure 1). The shared feeding habits and reliance of *A. pisum* and *D. citri* on Gram-negative obligate nutritional symbionts suggest that these Hemipterans may have a shared history of adaptations promoting close relationships with this group of microorganisms.

## Materials and methods

### Insects

*Diaphorina citri* were collected from a citrus grove in Ft. Pierce, FL (GPS coordinates 27.4389°N, 80.3356°W) and reared at the USDA, ARS, U.S. Horticultural Research Laboratory, Ft. Pierce, FL. *D. citri* were caged for feeding on *Murraya paniculata* for 2 years, then *Citrus macrophylla* for the following 2 years. Psyllids and plants were maintained under natural day length cycles. Adult *D. citri* were collected within 1 month of emergences, submerged in liquid nitrogen, and held at −80°C until nucleic acid preparation.

### Cloning and purification of insect nucleic acids

RNA purification and sequencing followed identical protocols as described in Reese et al. (2014). Total RNA was extracted using the Qiagen RNeasy Mini Kit, per manufacturer recommended protocol. mRNA purification utilized a poly-T oligo-attached magnetic beads, Conversion into cDNA done using the mRNA sequencing preparation kit from Illumina (part number 1004898). Sequencing of the cDNA was performed on an Illumina GAIIx sequencing system.

### Genome

The *D. citri* genome is available on National Center for Biotechnology Information (NCBI) under Genome Sequencing Project-RefSeq: Accession: PRJNA29447 ID: 29447 (http://www.ncbi.nlm.nih.gov/bioproject/PRJNA29447) and the I5K arthropod genome project (https://i5k.nal.usda.gov/Diaphorina_citri).

### Gene identification and annotation

The comprehensive list of *D. citri* genes related to immune defenses was curated by screening for immune genes within the *D. citri* draft genome version 1.1 previously annotated by the NCBI automated refseq eukaryote genome annotation pipeline (Hunter et al., 2014). Also included were published sequences produced by recent deep sequencing surveys of *D. citri* (Hunter et al., 2014; Vyas et al., 2015). Immune related genes of interest were selected based on previous surveys of arthropod immune pathways and immunological defenses (Gerardo et al., 2010; González-Santoyo and Córdoba-Aguilar, 2011; Buchon et al., 2014; Smith and Pal, 2014). In addition to screening published sequences, a manual search for *D. citri* immune related genes was performed to search for genes not identified in previous annotations and to check the validity of the automated pipeline annotations. The manual search was implemented by first locally blasting *D. citri* predicted transcripts against a locally curated list of immune related genes from *Drosophila melanogaster* (flybase.org), *A. pisum, Apis mellifera, Aedes aegypti* (vectorbase.org), *Bombyx mori*, and *Nilaparvata lugens* with the tblastx search algorithm. Geneious software (http://www.geneious.com, version 7.1.5) was used to conduct BLAST searches (Kearse et al., 2012) with results limited to hits with *e*-values of <1e^−5^. The identities of predicted transcripts with positive matches in the initial tblastx search were validated performing a BLAST search of all insect protein sequences in the NCBI nr database using blastp. Immune genes identified with the manual search were submitted to NCBI and sequences mislabeled by the automated annotation pipeline were updated.

### Phylogenetic tree

Phylogenetic analysis of Hdd11, glutathione s-transferases, and lysozymes, were created with Geneious tree builder (http://www.geneious.com, version 7.1.5). The trees were created by global alignment with free end gaps, identity cost matrix, Jukes-Cantor genetic distance model, and neighbor-joining tree building method. Sequences used are listed in supplemental tables: Hdd11 (Table S3), lysozymes (Table S4), and glutathione s-transferases (Table S5).

## Results and discussion

### Recognition proteins

#### Peptidoglycan recognition protein

Peptidoglycan recognition proteins (PGRPs) bind to the peptidoglycan in the bacterial cell and activate cellular signaling pathways, induce the melanization cascade, have amidase activity, or act an opsonization factors (Dziarski and Gupta, 2006). The structure of peptidoglycan is conserved between classes of bacteria with Gram-positive having L-lysine type peptidoglycan and Gram-negative bacteria and Gram-positive bacilli are made of Dap-type peptidoglycan in their respective cell walls (Schleifer and Kandler, 1972). The conserved nature of peptidoglycan aids in the efficiency of the insect immune response and prevents adaptations that would allow bacteria to avoid recognition. PGRPs are also highly conserved, being first identified in insects and subsequently identified in humans, mice, and other vertebrates (Kang et al., 1998).

There are two classes of PGRPs in insects: short cytostolic PGRP-Ss and long membrane-bound PGRP-Ls, both containing a 166 bp PGRP domain (IPR002502) located at the C-terminal region (Werner et al., 2000; Dziarski and Gupta, 2005). The number of PGRPs found in insects is diverse; for example, *D. melanogaster* has seven short and 10 long PGRPs, while *A. gambiae* has three short and four long PGRPs (Werner et al., 2000; Christophides et al., 2002). The diversity of PGRPs is associated with a variety of downstream responses. PGRP-SA, SD, and SC are activated by Lys-type peptidoglycan and subsequently cleave Spaetzle, inducing the Toll pathway (Michel et al., 2001; Gottar et al., 2002; Bischoff et al., 2004). PGRP-LC and SC1a activate phagocytosis of bacteria (Rämet et al., 2002; Garver et al., 2006). PGRP-LC in association with PGRP-LE recognize Dap-type peptidoglycan and activate the Imd pathway (Choe et al., 2002; Gottar et al., 2002; Werner et al., 2003; Takehana et al., 2004). PGRPs were not evident in the *D. citri* genome, though one gene coding for a lysM peptidoglycan-binding domain-containing protein was identified, though lacking the PGRP domain (Table 1). Despite being highly conserved among other holometabolous insects, they are absent from the genomes of at least two hemimetabolous insects, the pea aphid, *A. pisum*, and human louse, *Pediculus* spp., challenging the assumption that all insects possess relatively similar innate immune signaling pathways (Gerardo et al., 2010; Kim et al., 2011). The phylogenetic proximity of *D. citri* to *A. pisum*, both of the suborder Sternorrhyncha, implies that the absence of PGRP may be a shared trait among insects in this suborder. Though absent in these species, an investigation into the innate immune systems of the Hemipterans *N. lugens* and *R. prolixus* identified PGRPs, insinuating that the loss of PGRP is not a trait of all Hemiptera but specific to Sternorrhyncha (Bao et al., 2013; Mesquita et al., 2015).

**Table 1:** **Pattern recognition receptor genes**.

**Gene prediction**	**Gene ID**	**Accession**	**Scaffold**	**Exon**	**Orientation**	**Best match**	**Accession**	**Coverage (%)**	***e*-value**	**Identity (%)**
Down syndrome cell adhesion molecule-like protein Dscam2	LOC103522222	XM_008487323.1	NW_007384837.1	3	+	*Drosophila rhopaloa*	XP_016976232.1	99	5.00E-102	75
Down syndrome cell adhesion molecule-like protein Dscam2	LOC103519752	XM_008484838.1	NW_007380337.1	7	−	*Halyomorpha halys*	XP_014291200.1	38	3.00E-67	44
Down syndrome cell adhesion molecule-like protein Dscam2	LOC103518895	XM_017447974.1	NW_007379843.1	3	−	*Drosophila suzukii*	XP_016924697.1	83	9.00E-101	59
Down syndrome cell adhesion molecule-like protein Dscam2	LOC103518529	XM_017447838.1	NW_007379655.1	18	+	*Halyomorpha halys*	XP_014291201.1	96	0	47
Down syndrome cell adhesion molecule-like protein Dscam2	LOC103517118	XM_017447332.1	NW_007379177.1	38	−	*Tribolium castaneum*	XP_015836914.1	99	0	37
Down syndrome cell adhesion molecule-like protein Dscam2	LOC103516489	XM_017447077.1	NW_007379005.1	26	+	*Cimex lectularius*	XP_014244456.1	93	0	49
Down syndrome cell adhesion molecule-like protein Dscam2	LOC103506111	XM_017442707.1	NW_007377652.1	22	+	*Eufriesea mexicana*	XP_017766445.1	85	1.00E-142	36
Down syndrome cell adhesion molecule-like protein Dscam2	LOC103506112	XM_017442708.1	NW_007377652.1	23	−	Eufriesea mexicana]	XP_017766445.1	85	1.00E-142	36
Galectin-4-like	LOC103513039	XM_017445656.1	NW_007378347.1	6	−	*Eufriesea mexicana*	OAD54547.1	35	8.00E-06	31
Galectin-5-like	LOC103524901	XM_017449781.1	NW_007377561.1	4	+	*Amyelois transitella*	XP_013191099.1	46	1.00E-33	54
Galectin-5-like	LOC103506737	XM_008471139.2	NW_007377561.1	5	−	*Fopius arisanus*	XP_011310382.1	91	9.00E-30	40
32 kDa beta-galactoside-binding lectin-like	LOC103506738	XM_017442985.1	NW_007377695.1	7	−	*Zootermopsis nevadensis*	KDR14367.1	59	1.00E-51	36
E-selectin-like	LOC103509753	XM_008474381.2	NW_007377959.1	12	−	*Pediculus humanus corporis*	XP_002431650.1	74	0	57
Scavenger receptor class B member 1	LOC103511262	XM_008475980.1	NW_007378127.1	8	+	*Camponotus floridanus*	EFN74299.1	79	0	62
Scavenger receptor class B member 1-like	LOC103509988	XM_017444388.1	NW_007377986.1	10	+	*Diuraphis noxia*	XP_015379122.1	74	1.00E-137	52
Defense protein Hdd11	LOC103508820	XM_008473396.1	NW_007377872.1	3	+	*Cimex lectularius*	XP_014249583.1	39	6.00E-39	44

#### Gram-negative binding proteins

β-1,3-glucan recognition proteins (βGRPS), or Gram-negative binding proteins (GNBPs), are hemolymph-soluble PRRs that bind to the surface of Gram-positive bacteria or fungi. Binding results in the activation of serine proteases that initiate the Toll pathway, resulting in the production of antimicrobial peptides, or activating the prophenoloxidase melanization cascade (Roh et al., 2009; Wang et al., 2011). The structure of GNBP-1 and GNBP-3 consists of a C-terminal GNBP homology domain followed by a N-terminal β-1,3-glucan binding domain, while GNBP-2 lacks the C-terminal GNBP domain. GNBP-1 forms a complex with PGRP-SA, with both required to cleave Spaetzle and initiate an immune response via the Toll pathway (Gobert, 2003; Pili-Floury et al., 2004). GNBP-2 does not have glucanase activity, but binds to laminarin and lipoteichoic acid, and is associated with activating phenoloxidase (Jiang et al., 2004). GNBP-3, like GNBP -1, activates the Toll pathway in association with surface receptor binding (Gottar et al., 2006).

Gram-negative binding proteins and β-1,3-glucan recognition proteins were not found in *D. citri*, but are present in Hemipterans *A. pisum, N. lugens*, and *R. prolixus* (Ursic-Bedoya and Lowenberger, 2007; Gerardo et al., 2010; Bao et al., 2013). The presence of GNBP-1 in pea aphids is particularly interesting given that GNBP-1 in *Drosophila* does not function in the absence of PGRP-SA, which *A. pisum* lacks (Gobert, 2003; Gerardo et al., 2010). The absence of GNBPs in *D. citri* is unique, and may explain the high mortality of *D. citri* observed in response to entomopathogenic fungi in field and laboratory studies (Aubert, 1987; Avery et al., 2009, 2011).

#### DSCAM

Down syndrome cell adhesion molecule (DSCAM) is a pathogen recognition and embryonic development protein with a diversity of potential spliceforms (Schmucker and Chen, 2009). Alternative splicing of DSCAM pre-RNA results in a potential for 38,016 isoforms that may enhance the arthropod-pathogen recognition response (Watson et al., 2005). Alternate splicing is suspected to be influenced by pathogen activation of the Toll and Imd pathways (Dong et al., 2006, 2012a). There are two forms of DSCAM: a membrane-bound form in hemocytes, and a hemolymph soluble form.

Eight regions of repeated Ig domains similar to the gene structure of DSCAM were identified in *D. citri* (Table 1). Due to the highly variable nature of these regions, complete annotation of this gene was not completed, but it can be assumed that *D. citri* does possess at least one complete gene coding for DSCAM. The lack of PGRP and GNBP found in *D. citri* could indicate an increased need for DSCAM-initiated humoral immune recognition and defense against microorganisms. The absence of PGRPs and GNBPs necessary for Toll and Imd pathway activation in *D. citri* suggests that these immune pathways do not exclusively control the alternative splicing of DSCAM. It is also possible that DSCAM in *D. citri* is exclusively associated with neuronal function and development rather than immune function.

#### Other pattern recognition receptors

Galectins are a family of soluble, carbohydrate-binding proteins found in all metazoan organisms. They serve a number of functions, including cell adhesion, proliferation, migration, apoptosis, inflammation, and immunity (Hughes, 2001; Rabinovich et al., 2002). Insect galectins may act as PRRs, effectors, and aid hemocytes through agglutination and opsonization of bacteria (Baum et al., 2014).

Another diverse family of immune related binding proteins are the C-type lectins. C-type lectins are denoted as calcium-dependent carbohydrate-binding proteins, though they may or may not have calcium-binding or carbohydrate-binding regions, and thus the name is a misnomer. The structural motif of C-type lectins consists of a mannose-binding domain and act as PRRs (Zelensky and Gready, 2005; Tian et al., 2009). C-type lectins can be membrane-bound or extracellular. The function of C-type lectins is as diverse as those seen in galectins and has been implicated in prophenoloxidase activation, phagocytosis, and hemocyte proliferation (Yu and Kanost, 2003; Ling et al., 2008; Tian et al., 2009).

*D. citri* has a wide variety of potential recognition proteins, including C-type lectins and galectins (Table 1). Further investigations are needed to determine the functions of these proteins and their role in the *D. citri* immune response.

Animals have numerous leucine-rich repeat containing proteins (LRRs) that serve a variety of functions in cells, including acting as PRRs. In the Lepidopteran, *Manduca sexta*, a LRR, Leureptin, was found to bind to lipopolysaccharide and interact with hemocytes, potentially acting as an opsonization factor (Zhu et al., 2010). In *A. gambiae*, two LRRs, APL1, and LRIM1, aid in TEP1 binding to *Plasmodium* in a complement system (Fraiture et al., 2009). The *D. citri* genome annotation identified 46 LRRs with unknown functions (Table S1). Further transcriptional analysis of *D. citri* following immune challenge will elucidate if any of these proteins participate in immune functions.

**Figure 2:**
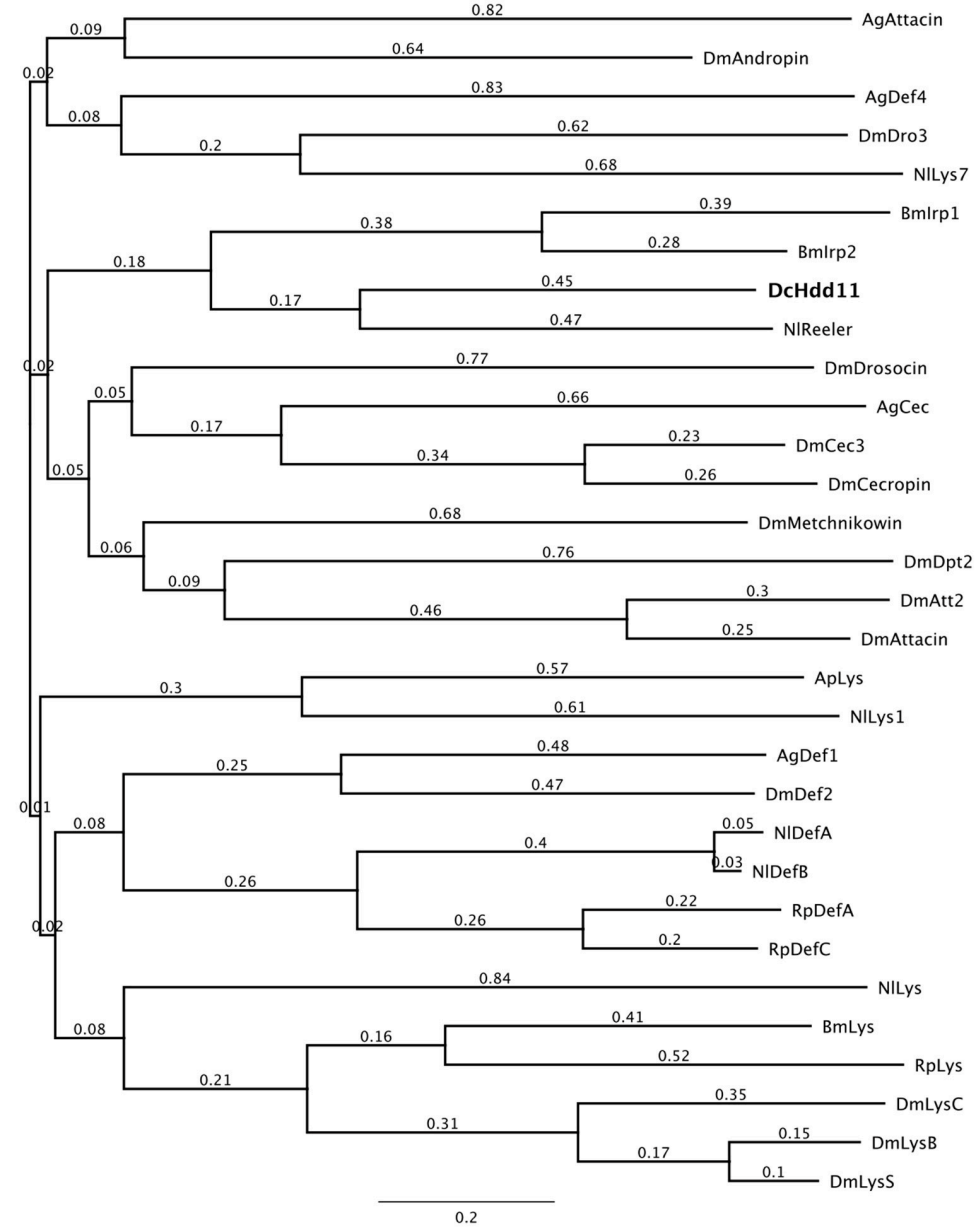
**A phylogenetic comparison of insect effectors with *D. citri* predicted defensive protein Hdd11 (XM_008473396.1)**. The phylogenetic tree was constructed using the Geneious (Version 7.1, Kearse et al., 2012) Tree Builder. Branch labels signify substitutions per site. Sequences used are listed in Table S3. Dm, *Drosophila melanogaster*; Ag, *Anopheles gambiae*, Ap, *Acyrthosiphon pisum*; Nl, *Nilaparvata lugens*; Bm, *Bombyx mori*; Dc, *Diaphorina citri*, Rp, *Rhodnius prolixus*.

Proteins containing a reeler domain are involved in the nodulation response, binding hemocytes to lipopolysaccharides, lipoteichoic acid, and beta-1,3 glucans, and can be involved in the phenoloxidase cascade (Gandhe et al., 2007; Bao et al., 2011). *D. citri* has one predicted sequence containing a reeler domain, defense protein Hdd11 (Table 1). A phylogenetic comparison of Hdd11 to effector molecules from other insects showed that this gene has sequence similarity with *N. lugens* Reeler (KC355218.1) and *B. mori* Immune response proteins (NM_001098349.1, NM_001257010.1) which contain a reeler domain (Figure 2).

### Immune signaling pathways

#### Toll

The Toll pathway is associated with the insect innate immune response to Gram-positive bacteria and fungi (Aggarwal and Silverman, 2008; Hetru and Hoffmann, 2009). Additionally, the Toll pathway in insects is involved in dorsal-ventral cellular patterning in embryonic development (Hashimoto et al., 1988). Microbial activation of the Toll pathway in *Drosophila* has been observed to activate the production of antimicrobial peptides and induce the propagation and differentiation of hemocytes (Valanne et al., 2011; Buchon et al., 2014).

The Toll receptor is membrane-bound, consisting of extracellular regions of leucine-rich repeats (LRRs) flanked by cysteine-rich domains, with the exception of Toll-9, which has only one cysteine-rich domain before the transmembrane domain (Tauszig et al., 2000; Imler and Hoffmann, 2001). The intracellular portion of the Toll receptor is a Toll/IL-1R (TIR) domain that connects with the downstream signaling proteins (Imler and Hoffmann, 2001).

Toll activation occurs when Spaetzle binds to the Toll receptor (Weber et al., 2003). Spaetzle is activated by proteolysis of its prodomain following microbial detection (Arnot et al., 2010). Proteolysis requires a Spaetzle-processing enzyme (SPE), which is activated by one of three signal cascades elicited in response to different classes of microorganisms. Two of these cascades are activated by Gram-positive-specific serine protease (Grass), and a third by the serine protease, Persephone (Ligoxygakis et al., 2002a; Gottar et al., 2006). Following Spaetzle binding to the Toll receptor, the intracellular Toll TIR domain binds directly to the heteromeric death domain containing proteins MyD88, Tube, and Pelle by the TIR domain on MyD88 (Sun et al., 2002; Moncrieffe et al., 2008). Signal transduction through these scaffold proteins leads to the phosphorylation and degradation of Cactus, which is bound to the NF-kappa-B-like transcription factors Dif or Dorsal (Kidd, 1992). Once separated from Cactus, Dif and Dorsal initiate transcription of antimicrobial peptide genes or other immune responses (Ip et al., 1993; Gross et al., 1996; Rutschmann et al., 2000).

Genes for six Toll receptors, one Spaetzle, and the intracellular signaling components MyD88, Pelle, Tube, Cactus, and Dorsal were identified in *D. citri* (Table 2). The role of Toll in the *D. citri* immune response is unclear, given that PGRP and GNBP genes associated with microbial recognition were not detected. Fungal detection receptors Persephone and Necrotic were also absent, which may explain the high mortality of *D. citri* in response to fungal pathogens (Aubert, 1987; Avery et al., 2009, 2011). The Toll pathway in *D. citri* is similar to that of *A. pisum*, which has a complete Toll pathway but lacks PGRPs. *A. pisum* has two genes coding for GNBPs that may function in fungal recognition. *A. pisum* also produce Thaumatins, a group of anti-fungal peptides, which were not detected in *D. citri* (Gerardo et al., 2010).

**Table 2:** **Toll pathway associated genes**.

**Gene prediction**	**Gene ID**	**Accession**	**Scaffold**	**Exon**	**Orientation**	**Best Match**	**Accession**	**Coverage**	***e*-value**	**Identity**
Protein spaetzle	LOC103511118	XM_008475831.2	NW_007378110.1	3	−	*Zootermopsis nevadensis*	KDR17073.1	39	2.00E-16	36
Protein toll	LOC103509078	XM_017444026.1	NW_007377899.1	3	−	*Cimex lectularius*	XP_014242709.1	99	0	62
Protein toll-like	LOC103511892	XM_017445172.1	NW_007378197.1	4	+	*Pediculus humanus corporis*	XP_002424101.1	87	6.00E-51	32
Protein toll-like	LOC103515901	XM_008480845.2	NW_007378866.1	3	+	*Polistes dominula*	XP_015181711.1	59	5.00E-12	28
Protein toll-like	LOC103518109	XM_017447692.1	NW_007379501.1	9	−	*Zootermopsis nevadensis*	KDR13629.1	63	6.00E-114	31
Toll-like receptor 3	LOC103507652	XM_008472152.1	NW_007377766.1	2	−	*Halyomorpha halys*	XP_014285049.1	86	9.00E-117	54
Toll-like receptor 3	LOC103508217	XM_017443652.1	NW_007377813.1	17	−	*Riptortus pedestris*	BAN20215.1	57	3.00E-90	40
Slit homolog 3 protein	LOC103511429	XM_008476154.2	NW_007378147.1	2	+	*Halyomorpha halys*	XP_014271067.1	88	0	67
insulin-like growth factor-binding protein complex acid labile subunit	LOC103515900	XM_008480843.2	NW_007378866.1	6	+	*Halyomorpha halys*	XP_014288082.1	71	3.00E-100	30
Toll-interacting protein B-like	LOC103516940	XM_008481931.1	NW_007379133.1	3	+	*Halyomorpha halys*	XP_014283422.1	60	2.00E-96	58
Serine/threonine-protein kinase pelle-like	LOC103510767	XM_017444717.1	NW_007378073.1	2	+	*Acyrthosiphon pisum*	XP_001943030.2	55	5.00E-139	49
Myeloid differentiation primary response protein MyD88	LOC103524770	XM_008489802.2	NW_007377556.1	8	−	*Cimex lectularius*	XP_014242274.1	62	6.00E-27	30
Protein Tube	LOC103523448	XM_008488489.1	NW_007400717.1	1	−	*Apis cerana*	XP_016910235.1	40	4.00E-17	43
NF-kappa-B inhibitor cactus	LOC103524536	XM_008489561.2	NW_007377540.1	4	+	*Halyomorpha halys*	XP_014275497.1	51	6.00E-46	37
Cactin	LOC103509881	XM_008474513.2	NW_007377974.1	10	−	*Bombus terrestris*	XP_003395486.1	55	3.00E-113	67
Embryonic polarity protein dorsal-like	LOC103524782	XM_017449739.1	NW_007377556.1	12	+	*Acromyrmex echinatior*	XP_011055463.1	44	1.00E-94	51

#### Imd and JNK

Insect recognition and systemic responses to Gram-negative bacteria are exclusively associated with the immune deficiency (Imd) pathway (Kleino and Silverman, 2014). The PRRs associated with the Imd pathway are the transmembrane PGRP-LC receptor and extracellular PGRP-LE, which work synergistically (Takehana et al., 2004). The intracellular signaling cascade is a complex of connector protein Imd that bind to FADD through their death domains and caspace-8 homolog DREDD. DREDD cleaves the N-terminal portion of NF-kappa-B transcription factor Relish and also Imd. Relish induces production of antimicrobial peptides (Hedengren et al., 1999). Cleaved and ubiquinated, Imd recruits TAK1 and TAB2 dimer, which subsequently phosphorylates Kenny/IKKb (Geuking et al., 2009). The Kenny/IKKb complex will either phosphorylate Relish, leading to nuclear translocation, or MKK7/hemipterous, which will lead to phosphorylation of Basket and c-Jun N-terminal kinase (JNK) (Geuking et al., 2009; Cao et al., 2015). JNK is a mitogen-activated protein kinase (MAPK) pathway involved in apoptosis, wound healing, and phagocytosis (Rämet et al., 2002; Mizutani et al., 2003).

The Imd pathway was mostly absent in *D. citri*, although TBK1 and the downstream JNK pathway was identified (Table 3), suggesting that alternative activation of JNK may occur. *R. prolixus, A. pisum*, and *P. humanus* also lack a portion of, or the entire Imd pathway (Gerardo et al., 2010; Kim et al., 2011; Mesquita et al., 2015). Absence of the Imd pathway is possibly associated with the evolution of the relationships all Sternorrhyncha share with bacterial endosymbionts (Altincicek et al., 2008). Sternorrhynchan endosymbionts are primarily Gram-negative bacteria, though some harbor yeast-like symbionts, thus absence of the Imd pathway may facilitate colonization of the insect host, an adaptation that allowed these insects to feed on nutritionally-poor plant phloem or xylem. Facultative symbionts may benefit insects by providing antimicrobial or parasitoid defense, expanding host range, or expanding heat tolerance (Montllor et al., 2002; Oliver et al., 2003; Scarborough et al., 2005; McLean et al., 2011; Ye et al., 2013). The benefits of the facultative symbionts are suspected to be strong drivers of speciation in this group (Tsuchida et al., 2004, 2011; Cordaux et al., 2011).

**Table 3:** **Imd and JNK associated genes**.

**Gene prediction**	**Gene ID**	**Accession**	**Scaffold**	**Exon**	**Orientation**	**Best match**	**Accession**	**Coverage (%)**	***e*-value**	**Identity (%)**
Serine/threonine-protein kinase TBK1	LOC103512606	XM_008477381.2	NW_007378295.1	10	−	*Acyrthosiphon pisum*	XP_001946184.1	69	0	64
Putative inhibitor of apoptosis	LOC103506717	XM_008471116.2	NW_007377694.1	6	−	*Lygus lineolaris*	ADK56128.1	65	2.00E-100	45
Apoptosis inhibitor 5-like	LOC103505580	XM_008469919.2	NW_007377619.1	8	−	*Microplitis demolitor*	XP_008545864.1	86	6.00E-111	56
Apoptosis inhibitor 5-like	LOC103520736	XM_017448611.1	NW_007381407.1	5	−	*Zootermopsis nevadensis*	KDR14692.1	97	6.00E-161	49
TP53-regulated inhibitor of apoptosis 1-like	LOC103505407	XM_008469737.2	NW_007377609.1	3	−	*Microplitis demolitor*	XP_008548768.1	23	5.00E-29	61
TNF receptor-associated factor 4	LOC103507530	XM_008472010.2	NW_007377755.1	7	+	*Cimex lectularius*	XP_014244337.1	72	0	62
TNF receptor-associated factor 6-like	LOC103510016	XM_017444539.1	NW_007377463.1	5	+	*Cyphomyrmex costatus*	KYN07335.1	54	3.00E-38	38
TNF receptor-associated factor 6-like	LOC103518718	XM_008483799.2	NW_007379755.1	3	+	*Papilio xuthus*	KPI91118.1	85	1.00E-39	25
TNF receptor-associated factor 6-like	LOC103518719	XM_008483801.1	NW_007379755.1	2	+	*Megachile rotundata*	XP_012148205.1	63	1.00E-27	41
Stress-activated protein kinase JNK	LOC103508970	XM_017443978.1	NW_007377889.1	6	+	*Zootermopsis nevadensis*	KDR18179.1	92	2.00E-143	98

#### JAK/STAT

The Janus kinase/signal transducer activator of transcription (JAK/STAT) pathway functions in embryonic development, and participates in humoral and cellular immune responses to bacteria, fungi, parasitoids, and viruses (Sorrentino et al., 2004; Dostert et al., 2005; Cronin et al., 2009; Souza-Neto et al., 2009; Dong et al., 2012b; Kingsolver et al., 2013). Unlike the Toll and Imd pathways, which are activated in response to PRR binding to PAMPs, the JAK/STAT pathway is activated by cytokines (Harrison et al., 1998; Agaisse and Perrimon, 2004). Extracellular cytokine binding to a pair of transmembrane cytokine receptors activates a JAK proteins on the intracellular region, which phosphorylate Domeless (Brown et al., 2001). STAT92E (STAT) then binds to Domeless and becomes phosphorylated (Hou et al., 2002). Phosphorylated STATs dimerize and pass into the nucleus, where they act as transcription factors (Yan et al., 1996). The primary components of the JAK/STAT pathway were identified in *D. citri* (**Table 5**).

JAK/STAT activation in response to stress or immune challenge increases the expression of Tot and thioester-containing proteins (Teps), in *Drosophila* (Lagueux et al., 2000; Agaisse et al., 2003). Tot proteins do not have direct antibacterial properties, but are associated with the general stress response and may function as transcription factors for cellular protection or repair (Ekengren and Hultmark, 2001). Neither Tep nor Tot were identified in *D. citri* (Table 4).

**Table 4:** **Jak/STAT associated genes**.

**Gene prediction**	**Gene ID**	**Accession**	**Scaffold**	**Exon**	**Orientation**	**Best match**	**Accession**	**Coverage (%)**	***e*-value**	**Identity (%)**
Tyrosine-protein kinase JAK2	LOC103524755	XM_017449726.1	NW_007377555.1	7	+	*Tribolium castaneum*	XP_008196394.1	76	3.00E-142	45
Signal transducer and activator of transcription 5B	LOC103509325	XM_008473935.2	NW_007377923.1	14	+	*Ceratina calcarata*	XP_017889583.1	90	0	58
E3 SUMO-protein ligase PIAS3-like	LOC103509716	XM_017444288.1	NW_007377956.1	12	−	*Zootermopsis nevadensis*	KDR14768.1	46	2.00E-84	49
Suppressor of cytokine signaling 5	LOC103515668	XM_008480608.2	NW_007377487.1	1	+	*Zootermopsis nevadensis*	KDR10999.1	54	1.00E-135	57
Serine/threonine-protein kinase A-Raf-like	LOC103523853	XM_008488867.1	NW_007424942.1	1	−	*Habropoda laboriosa*	XP_017789472.1	100	2.00E-90	78

JAK/STAT is also involved in the differentiation and proliferation of hemocytes (Minakhina et al., 2011). Proliferation of hemocytes is activated by STAT92E, promoting the expression of Raf MAPK, while Raf has been seen to interact with Hopscotch independent of STAT92E, leading to the proliferation of hemocytes (Luo et al., 2002). One Raf-like protein gene was identified in *D. citri* (Table 5). Further investigation is needed to expand current understanding of the JAK/STAT pathway in *D. citri* immune responses.

**Table 5:** **RNAi associated genes**.

**Gene prediction**	**Gene ID**	**Accession**	**Scaffold**	**Exon**	**Orientation**	**Best match**	**Accession**	**Coverage (%)**	***e*-value**	**Identity (%)**
Argonaute-2	LOC103507599	XM_008472091.1	NW_007377764.1	11	+	*Halyomorpha halys*	XP_014287705.1	79	0	88
Dicer homolog 3-like	LOC103518839	XM_008483925.1	NW_007379804.1	7	−	*Papilio polytes*	XP_013149103.1	66	9.00E-24	38
endoribonuclease Dcr-1-like	LOC103506500	XM_008470888.1	NW_007377676.1	7	−	*Cephus cinctus*	XP_015607218.1	96	0	44

### Immune response

#### RNAi

Toll, Imd, and JAK/STAT pathways are involved in viral recognition and response; however, RNA interference (RNAi) is the primary antiviral defense system used by insects. Viral double-stranded RNAs (dsRNA) are detected and used as templates for recognition and detection of the same viral RNA. Dicer-2, in conjunction with R2D2, detects viral RNA and cleaves it into short 21–22 bp segments (Elbashir et al., 2001; Liu et al., 2003). The short dsRNA segment is bound to a RNA-induced silencing complex (RISC) of Argonaut and C3P0, and the passenger strand is degraded (Hammond et al., 2001). The RISC complex uses the remaining guide strand to bind to complementary viral mRNAs, cleave them, and inhibit viral replication. Silencing insect genes using RNAi is a promising new tool for *D. citri* management (El-Shesheny et al., 2013; Killiny et al., 2014). The primary components of the RNAi system, Argonaut, Dicer, and SID-1, were identified in *D. citri* (Table 5). Although C3P0 was not identified in *D. citri*, Argonaut-2 can function as an endonuclease without the presence of other associated RNAi proteins (Rand et al., 2004). In contrast, the absence of R2D2 is surprising, given that it is required for proper loading of double-stranded RNA into Dicer (Liu et al., 2006).

#### Melanization and clotting

Melanization of wound sites and invading microorganisms is a primary terminal component of the arthropod immune system and is often used to quantify immune response (Cerenius et al., 2008; Tang, 2009). Melanization is activated by both the Toll and Imd pathways (Takehana et al., 2002; Park et al., 2007). Activation of prophenoloxidase (PPO) by serine proteases produces defensive cytotoxic quinones, and aids in phagocytosis and blood clotting (Theopold et al., 2004; Liu et al., 2007; Zhao et al., 2007). Due to the potential for self-harm by quinones, PPO activation is a highly regulated system that produces a localized and rapid response (Ligoxygakis et al., 2002b; Michel et al., 2005; Nappi et al., 2005). *D. citri* has the components of the PPO pathway (**Table 7**). Melanization occurs rapidly in extracted hemolymph and following cuticular puncture (personal observation). In contrast, *A. pisum* does not exhibit a strong melanization response to cuticle punctures (Altincicek et al., 2008). Further investigations will determine the role of PPO genes in response to pathogen infection.

Melanization is a major component of wound healing in insects, but it is not necessary for clotting. A group of proteins called hemolectins, or hemocytins, and Fondue, participate in clotting and inhibition of bacterial invasion (Lesch et al., 2007). Hemocytins, including von Willebrand factors and discoidins, have domains homologous to vertebrate clotting factors (Kotani et al., 1995). Knockdown of hemolectins reduces clotting ability but does not affect survival of bacterial infections, even in cases of oral exposure to the pathogen (Lesch et al., 2007; Chang et al., 2012). Hemocyins also contribute to nodulation after pathogen exposure, aiding in cellular immune responses (Arai et al., 2013). We identified three hemocytins, although the *Drosophila* clotting protein Fondue was not identified (Table 6).

**Table 6:** **Clotting and melanization associated genes**.

**Gene prediction**	**Gene ID**	**Accession**	**Scaffold**	**Exon**	**Orientation**	**Best match**	**Accession**	**Coverage (%)**	***e*-value**	**Identity (%)**
Proclotting enzyme-like	LOC103520132	XM_017448420.1	NW_007380658.1	7	+	*Diuraphis noxia*	XP_015375315.1	61	0	62
Serine protease snake-like	LOC103516536	XM_008481505.1	NW_007379018.1	3	−	*Harpegnathos saltator*	XP_011141714.1	94	1.00E-18	42
Serine protease snake-like	LOC103506878	XM_008471301.1	NW_007377706.1	7	+	*Pediculus humanus corporis*	XP_002422611.1	78	6.00E-68	43
Serine protease snake-like	LOC103510170	XM_008474816.2	NW_007378002.1	8	−	*Pediculus humanus corporis*	XP_002422611.1	72	3.00E-57	49
Serine protease snake-like	LOC103512902	XM_008477687.2	NW_007378328.1	8	−	*Pediculus humanus corporis*	XP_002422611.1	66	5.00E-43	48
Serine protease snake-like	LOC103516535	XM_017447093.1	NW_007379018.1	6	−	*Cerapachys biroi*	XP_011341348.1	74	2.00E-75	44
Leukocyte elastase inhibitor A-like	LOC103524275	XM_008489289.1	NW_007377527.1	6	+	*Nilaparvata lugens*	AGK40932.1	69	6.00E-123	73
Serpin-Z1C-like	LOC103518206	XM_017448807.1	NW_007377443.1	19	−	*Anopheles sinensis*	KFB40965.1	95	2.00E-84	44
Serpin B7-like	LOC103507231	XM_008471684.1	NW_007377734.1	3	−	*Tribolium castaneum*	XP_969900.1	88	4.00E-26	41
Phenoloxidase 2-like	LOC103506840	XM_017443045.1	NW_007377702.1	9	+	*Apis cerana cerana*	AFU83105.1	78	7.00E-95	62
Phenoloxidase 2-like	LOC103506841	XM_017443046.1	NW_007377702.1	5	−	*Vollenhovia emeryi*	XP_011863706.1	93	5.00E-109	69
Hemocytin-like	LOC103508987	XM_017443986.1	NW_007377890.1	52	−	*Cimex lectularius*	XP_014260068.1	88	2.00E-145	39
Hemocytin-like	LOC103520268	XM_017448454.1	NW_007380802.1	13	−	*Zootermopsis nevadensis*	KDR23192.1	85	8.00E-167	54
Hemocytin-like	LOC103508986	XM_017443985.1	NW_007377890.1	10	+	*Cimex lectularius*	XP_014260068.1	77	4.00E-159	43
Multiple epidermal growth factor-like domains protein 10	LOC103510125	XM_017444457.1	NW_007377996.1	11	−	*Bombyx mori*	XP_012550133.1	86	7.00E-77	33

#### Antimicrobial peptides

Antimicrobial peptides (AMPs) are produced by all organisms, including bacteria, as a component of the innate immune system (Hancock and Diamond, 2000). These peptides are generally short, <100 residues long, yet they are diverse and have rapid rates of evolution. Evidence of this is apparent on the Antimicrobial Peptide Database, which contains over 2100 unique sequences [http://aps.unmc.edu/AP/main.php, (Wang et al., 2009)]. In insects, AMPs are often produced continuously to provide basal immunity against invading pathogens and to manage endosymbiont populations. They are also induced in response to the presence of microorganisms (Login et al., 2011). Most insects produce a wide variety of AMPs, often simultaneously, to ensure that the invading microorganism is eliminated and possibly to reduce the development of AMP resistance (Dobson et al., 2013). AMPs generally function by disrupting the cell membrane of bacterial cells due to their cationic and amphipathic structure (Brown and Hancock, 2006). Antifungal peptides, including thaumatins, such as those produced by aphids, were also absent (Gerardo et al., 2010). The lack of a diverse pool of antimicrobial peptides in *D. citri* as compared with other Hemipterans, *Drosophila*, and holometabolous insects, suggests that their absence in *D. citri* is the result of gene loss rather than a basal state.

Four lysozymes genes were present in *D. citri* (Table 7). Lysozymes are a specific group of cell wall-disrupting antimicrobial proteins that defend against bacteria and fungus found in both vertebrates and invertebrates. There are two types of lysozymes found in insects, i- and c-type, both of which have been observed to be involved in digestion and immune defenses, indicating that function is species specific or related to tissue level expression rather than associated with type (Mohamed et al., 2016). A comparison of lysozyme genes identified in *D. citri* with classified lysozymes in other insects indicates that two are likely c-type and one is i-type (Figure S1). The fourth lysozyme does not align with other lysozyme sequences and may be misannotated.

**Table 7:** **Effector genes**.

**Predicted gene**	**Gene ID**	**Accession**	**Scaffold**	**Exon**	**Orientation**	**Best match**	**Accession**	**Query**	***e*-value**	**Identity**
								**Cover (%)**		**(%)**
Defense protein Hdd11	LOC103508820	XM_008473396.1	NW_007377872.1	3	+	*Cimex lectularius*	XP_014249583.1	39	6.00E-39	44
Lysozyme-like	LOC103515209	XM_017446560.1	NW_007378726.1	5	−	*Diuraphis noxia*	XP_015370545.1	22	3.00E-32	62
Lysozyme-like	LOC103515655	XM_008480591.1	NW_007378815.1	10	+	*Acyrthosiphon pisum*	XP_001946402.2	32	0.001	31
Lysozyme-like	LOC103509720	XM_017444286.1	NW_007377956.1	6	+	*Papilio polytes*	XP_013149147.1	41	2.00E-21	47
putative lysozyme-like	LOC103510894	XM_008475598.1	NW_007377467.1	7	+	*none*				
Chitinase-3-like	LOC103510725	XM_008475412.1	NW_007378070.1	9	−	*Nilaparvata lugens*	AJO25037.1	66	4.00E-112	46
Probable chitinase 3	LOC103522082	XM_017448986.1	NW_007377515.1	27	−	*Trachymyrmex zeteki*	KYQ48585.1	91	0	48
Probable chitinase 3	LOC103521103	XM_017448708.1	NW_007381920.1	9	−	*Cimex lectularius*	XP_014241974.1	97	0.00E+00	60
Chitinase-like protein EN03	LOC103523836	XM_017449611.1	NW_007377444.1	6	+	*Halyomorpha halys*	XP_014277588.1	83	1.00E-153	53
Chitinase-like protein EN03	LOC103509630	XM_008474254.2	NW_007377949.1	4	+	*Halyomorpha halys*	XP_014277588.1	66	8.00E-45	53
Endochitinase-like	LOC103505867	XM_008470235.2	NW_007377449.1	17	−	*Poophilus costalis*	AFW03959.1	85	0.00E+00	74
Endochitinase-like	LOC103505882	XM_008470257.1	NW_007377449.1	3	−	*Cerapachys biroi*	EZA61225.1	99	2.00E-56	67
Acidic mammalian chitinase-like	LOC103510734	XM_008475421.1	NW_007378070.1	2	−	*Nilaparvata lugens*	AJO25037.1	96	2.00E-28	48
Acidic mammalian chitinase-like	LOC103518855	XM_008483937.2	NW_007379812.1	3	−	*Lucilia cuprina*	KNC31075.1	57	4.00E-10	42
Chitotriosidase-1-like	LOC103516792	XM_008481777.1	NW_007379091.1	5	+	*Lygus hesperus*	ANC95004.1	75	2.00E-29	45
Dual oxidase	LOC103506134	XM_017442724.1	NW_007377654.1	28	+	*Cimex lectularius*	XP_014254214.1	89	0	79
Dual oxidase-like	LOC103521505	XM_017448835.1	NW_007382726.1	12	−	*Anasa tristis*	AFK29281.1	96	0	85
Dual oxidase maturation factor 1-like	LOC103507952	XM_008472473.2	NW_007377456.1	7	+	*Acyrthosiphon pisum*	XP_001949862.1	58	4.00E-68	63
Nitric oxide synthase, salivary gland-like	LOC103521869	XM_008486976.1	NW_007383556.1	2	+	*Neodiprion lecontei*	XP_015516187.1	98	4.00E-49	79
nitric oxide synthase, salivary gland-like	LOC103509974	XM_017444384.1	NW_007377981.1	3	−	*Apis mellifera*	XP_016766542.1	83	7.00E-45	%

#### Nitric oxygen synthase

Invertebrate nitric oxides, produced by nitric oxide synthase (NOS), participate in neuron metabolism and immune response. Nitric oxides are highly diffusible and can cause reactions at very low concentrations. High concentrations of nitric oxide are cytotoxic to both microorganisms and the organism producing the NO (Kang et al., 1998; Colasanti et al., 2002). Insect NOS is upregulated in response to Gram-negative bacteria, fungi, and plasmodium parasites (Luckhart et al., 1998; Foley and O'Farrell, 2003). Two sequences were identified as a partial NOS in *D. citri* (Table 7). *A. aegypti, A. mellifera*, and *T. castaneum* all possess a single *NOS* gene, which suggests that the partial NOS sequences observed in *D. citri* may be portions of the complete gene (McTaggart et al., 2009).

#### Chitinase

Insects produce a variety classes of chitinases involved in molting, digestion, and cell proliferation following immune challenge (Arakane and Muthukrishnan, 2009). *Anopheles gambiae* have two type V chitinases that are secreted into the hemolymph following immune challenge and assist in the proliferation or recruitment of hemocytes and phagocytosis of invading bacteria (Shi and Paskewitz, 2004). Interestingly, these chitinase-like proteins are upregulated in the presence of bacteria but not fungi, although fungi have chitin cell walls (Shi and Paskewitz, 2004). Several chitinase-like proteins were predicted in *D. citri* (Table 7). The majority of these proteins are likely involved in molting, as *D. citri* do no feed on chitinous material and do not produce a peritrophic matrix.

#### Detoxification

Glutathione-s-transferases (GST) are a family of detoxification enzymes found in insects and almost all animals. GSTs detoxify exogenous compounds, including plant and microbial toxins, and pesticides (Kostaropoulos et al., 2001; Ortelli et al., 2003). In addition to detoxification, GSTs function in response to oxidative stress. Reactive oxygen compounds produced in response to microbial challenge are also cytotoxic to host tissues (Ranson and Hemingway, 2005). Insect GSTs were originally classified into type-1 and type-2, but further studies have broken these into delta, epsilon, omega, theta, and zeta groups (Ranson and Hemingway, 2005).

Ten GSTs were identified in *D. citri* (Table S2). In a phylogenetic comparison to identify GST group, two sequences aligned with microsomal, two with epsilon, two with delta, and three with sigma type GSTs (Figure S2). One *D. citri* GST did not align to any classified GSTs used in the analysis. No *D. citri* GSTs showed similarity to zeta, omega, or theta types. Kumar et al. (2003) reported that impaired GST function in a strain of *A. gambiae* reduced their ability to acquire *Plasmodium* parasites due to the oxidative stress inducing conditions favorable to melanin encapsulation. In *D. citri*, induction of high oxidative stress may inhibit acquisition of *C*Las, thus GSTs could be a target for future research to prevent or reduce *C*Las transmission. Further investigations will determine the role of these GST genes in *D. citri* immune function and pesticide detoxification.

## Conclusions

Current research on the innate immune system of insects has primarily focused on *Drosophila* and human disease vectors such as *Anopheles* mosquitoes. Only recently has the immune function in hemimetabolous insects, such as *A. pisum, N. lugens*, and *P. humanus* been explored (Ursic-Bedoya and Lowenberger, 2007; Gerardo et al., 2010; Kim et al., 2011; Bao et al., 2013). Interestingly, *A. pisum, R. prolixus*, and *P. humanus* have reduced immune systems as compared with holometabolous insects, while the *N. lugens* immune system appears similar to that of higher-order insects (Ursic-Bedoya and Lowenberger, 2007; Gerardo et al., 2010; Kim et al., 2011; Bao et al., 2013). Predicting the immune system defenses of *D. citri* is challenging, given the disparity among known Hemipteran immune genes. Annotation of the *D. citri* draft genome indicates that *D. citri* has a reduced innate immune system. In particular, genes related to the recognition of invading microorganisms, the Imd signaling pathway, and most antimicrobial peptides were absent. Although most insect genomes available for comparison are distantly related, and immune genes have high rates of evolution, these genes were identified in *N. lugens*. Furthermore, *Drosophila* gene sequences were used to identify immune genes in even more distantly-related arthropods, such as *Ixodes scapularis* (Schmid-Hempel, 2005; Bao et al., 2013; Smith and Pal, 2014). It is unlikely that the absence of these genes in *D. citri* is due to the high sequence divergence observed among immune genes.

*D. citri* may have a limited capacity for recognizing and eliminating the Gram-negative α–proteobacterial plant pathogen, *Candidatus* Liberibacter asiaticus, due to the absence of an Imd pathway (Kleino and Silverman, 2014). Although missing the pathway, *C*Las titers may decline over time in adult *D. citri* (Pelz-Stelinski et al., 2010). The reduction in titer could imply that alternate immune pathways or defenses are used by *D. citri*, such as cellular immune responses. Nymphs acquire *C*Las more efficiently than adults, suggesting that immune response may vary with life stage (Inoue et al., 2009; Pelz-Stelinski et al., 2010).

Altincicek et al. (2008) proposed three hypotheses for the reduced immune system found in *A. pisum*: *A*. *pisum* encounter few pathogenic microorganisms in their diet; energy that would be used toward an immune response is allocated to reproductive effort; or the reduced immune system is due to, or the result of, *A. pisum's* associated obligate and facultative bacterial symbionts.

The hypothesis that the *A. pisum* reduced immune system is a function of diet is based on comparisons to insects such as *Drosophila*, which live and feed on decaying materials, are exposed to more microorganisms, and have more robust innate immune systems. Insects that feed on phloem are exposed to far fewer microorganisms, thus innate immune defenses are less important (Altincicek et al., 2008). This hypothesis is unlikely, as *Sitophilus* weevil juvenile stages develop within the sterile interior of cereal grains but exhibit robust immune defenses (Anselme et al., 2008). Additionally, *A. pisum* can acquire lethal Pseudomonas syringae, a pathogenic epiphyte, from leaf surfaces when probing, indicating that their reduced immune capabilities are not directly related to their feeding habits or environment (Stavrinides et al., 2009).

Immune responses are costly, and trade-offs occur between immune response and reproductive effort (Sheldon and Verhulst, 1996). Evidence of the reproductive cost in insects is apparent, where after an immune challenge, hatch rate and reproductive attempts decrease, as in mosquitoes (Contreras-Garduño et al., 2014). Time of immune challenge can also impact the allocation away from immune response to reproductive effort, evident in locusts, where newly-emerged adults produce eggs soon after immune challenge, while older adults do not, indicating an age-related immune focus (Blanford and Thomas, 2001). Specificity of reproductive costs due to immune challenge occur in *A. pisum*. Exposure to fungi reduces fecundity, while exposure to Gram-negative bacteria does not (Barribeau et al., 2014). Sex can also impact the degree of immune response, as in *Gryllus texensis*, where sexually mature females maintain or display an increase in immunocompetence, compared to a decrease among sexually active males (Adamo et al., 2001).

The absence of Imd pathways and antimicrobial peptides in *A. pisum* and *D. citri* may be associated with the complex relationships these insects have with bacterial symbionts. Primary symbionts facilitate exploitation of niche resources that are otherwise unavailable to their insect host. Endosymbionts may share metabolic pathways to produce essential amino acids lacking in the insect diet, such as *Candidatus* Carsonella ruddii and *Buchnera aphidicola* symbionts of psyllids and aphids, respectively (Wilson et al., 2006; Dahan et al., 2015). They may also aid in the breakdown of cellulose into accessible sugars, as in the complexes of bacteria and yeast found in termite guts. Secondary symbionts are not essential to an insect's survival, but can serve beneficial functions, such as increasing heat tolerance, providing protection from parasitoids, or aiding in metabolism during periods of nutritional stress (Montllor et al., 2002; Oliver et al., 2003; Su et al., 2014). Secondary symbionts are also involved in insect immunity (Eleftherianos et al., 2013). The *A. pisum* secondary symbiont, *Regiella aphidicola*, provides protection against the entomopathogenic fungus, *Pandora neoaphidis* (Scarborough et al., 2005; Oliver et al., 2008). *Wolbachia*, reported in *D. citri*, are bacteria known to activate or prime the immune system of mosquitoes, providing an increased basal level of immunity and resistance (Moreira et al., 2009). *Candidatus* Proftella armatura occur in *D. citri* bacteriocytes and produce cytotoxic polyketide toxins (Nakabachi et al., 2013). Endosymbionts that escape the bacteriocyte and infect surrounding tissues may silence AMP production, as in *Sitophilus* beetles. Toxins produced by Proftella may be associated with regulating endosymbionts as an alternative to insect-produced AMPs (Login et al., 2011).

The absence of putative essential immune genes in *D. citri* supports what may be a common feature among the Hemiptera. Altincicek et al. (2008) discussed three hypotheses to explain the reduced immune system found in aphids, the most probable being that the reduced immune defenses aided in the evolution of complex symbiotic relationships with bacteria. All sap-feeding Hemiptera require symbiotic bacteria or fungi that participate in the production of essential amino acids absent in these insects' diets. Whether the loss of immune function allowed for the colonization of these beneficial bacteria, or the introduction of these bacteria led to the loss of immune function, is not known. This adaptation likely opened new niches for sap-feeding insects and acted as a driver in their speciation. Secondarily, some symbionts provide *A. pisum* with protection from parasitoids or fungi, increasing the insect' fitness to ensure their passage to the next generation. The dynamics of these relationships are poorly understood. Knowledge of these interactions is primarily based on studies of *A. pisum*, an insect with unusual reproductive behavior that may not be truly representative of all Hemiptera. Our observation that *D. citri* also lack the Imd pathway offers another model organism for investigating the effect of a reduced immune system on insect survival, and the contribution of symbionts to insect immunity. Finally, immune reduction in *D. citri* may contribute to their competence as vectors of *C*Las by allowing this pathogen to invade the insect freely, thus facilitating transmission.

## Author contributions

All authors contributed equally to this work. AA conducted the gene search and secondary BLAST survey and wrote the main paper. WH led the genome sequencing. All authors discussed the results and implications and commented on the manuscript at all stages.

## Funding

The Florida Citrus Research and Development Foundation, Inc, 2011 (Project # 05-021). Supported by grants from: USDA-ARS 2010–2011. The Florida Citrus Production Research Advisory Council, 2008. The BioScience Division, Los Alamos National Laboratory, NM, 2011–2012. The Citrus Research Board, 2012.

### Conflict of interest statement

The authors declare that the research was conducted in the absence of any commercial or financial relationships that could be construed as a potential conflict of interest.

## Supplementary material

Figure S1 **A phylogenetic comparison of *D. citri* predicted lysozymes with categorized lysozymes form other insects**. The phylogenetic tree was constructed using the Geneious (Version 7.1, Kearse et al., 2012) Tree Builder. Branch labels signify substitutions per site. Sequences used are listed in Table S4. Aa, *Aedes aegyptii*; Ag, *Anopheles gambiae*; Nl, *Nilaparvata lugens*; Dc, *Diaphorina citri*.

Figure S2 **A phylogenetic comparison *D. citri* glutathione s-transferases with categorized GSTs from other insects**. The phylogenetic tree was constructed using the Geneious (Version 7.1, Kearse et al., 2012) Tree Builder. Branch labels signify substitutions per site. Sequences used are listed in Table S5. Dm, *Drosophila melanogaster*; Ap, *Acyrthosiphon pisum*; Nl, *Nilaparvata lugens*; Bm, *Bombyx mori*; Dc, *Diaphorina citri*; Tm, *Tenebrio molitor*.

Table S1 **Leucine rich repeat containing protein genes**.

Table S2 **Free radical defense associated genes**.

Table S3 **Sequences used to create the phylogenetic tree in Figure 2**.

Table S4 **List of sequences used in Figure S1**.

Table S5 **List of sequences used in Figure S2**.
