# Supplementary material for: Annotation of the Asian Citrus Psyllid Genome Reveals a Reduced Innate Immune System
Source: Front Physiol. 2016 Nov 29;7:570. doi: 10.3389/fphys.2016.00570 (PMC5126049; doi:10.3389/fphys.2016.00570)
Supplement: Figure S1 — A phylogenetic comparison of D. citri predicted lysozymes with categorized lysozymes form other insects. The phylogenetic tree was constructed using the Geneious (Version 7.1, Kearse et al., 2012) Tree Builder. Branch labels signify substitutions per site. Sequences used are listed in Table S4. Aa, Aedes aegyptii; Ag, Anopheles gambiae; Nl, Nilaparvata lugens; Dc, Diaphorina citri. [file DataSheet1.DOCX]

Figure S1. A phylogenetic comparison of *D. citri* predicted lysozymes with categorized lysozymes form other insects. The phylogenetic tree was constructed using the Geneious (Version 7.1, Kearse et al., 2012) Tree Builder. Branch labels signify substitutions per site. Sequences used are listed in Table S4. Aa, *Aedes aegyptii*; Ag, *Anopheles gambiae*; Nl, *Nilaparvata* *lugens*; Dc, *Diaphorina citri*.


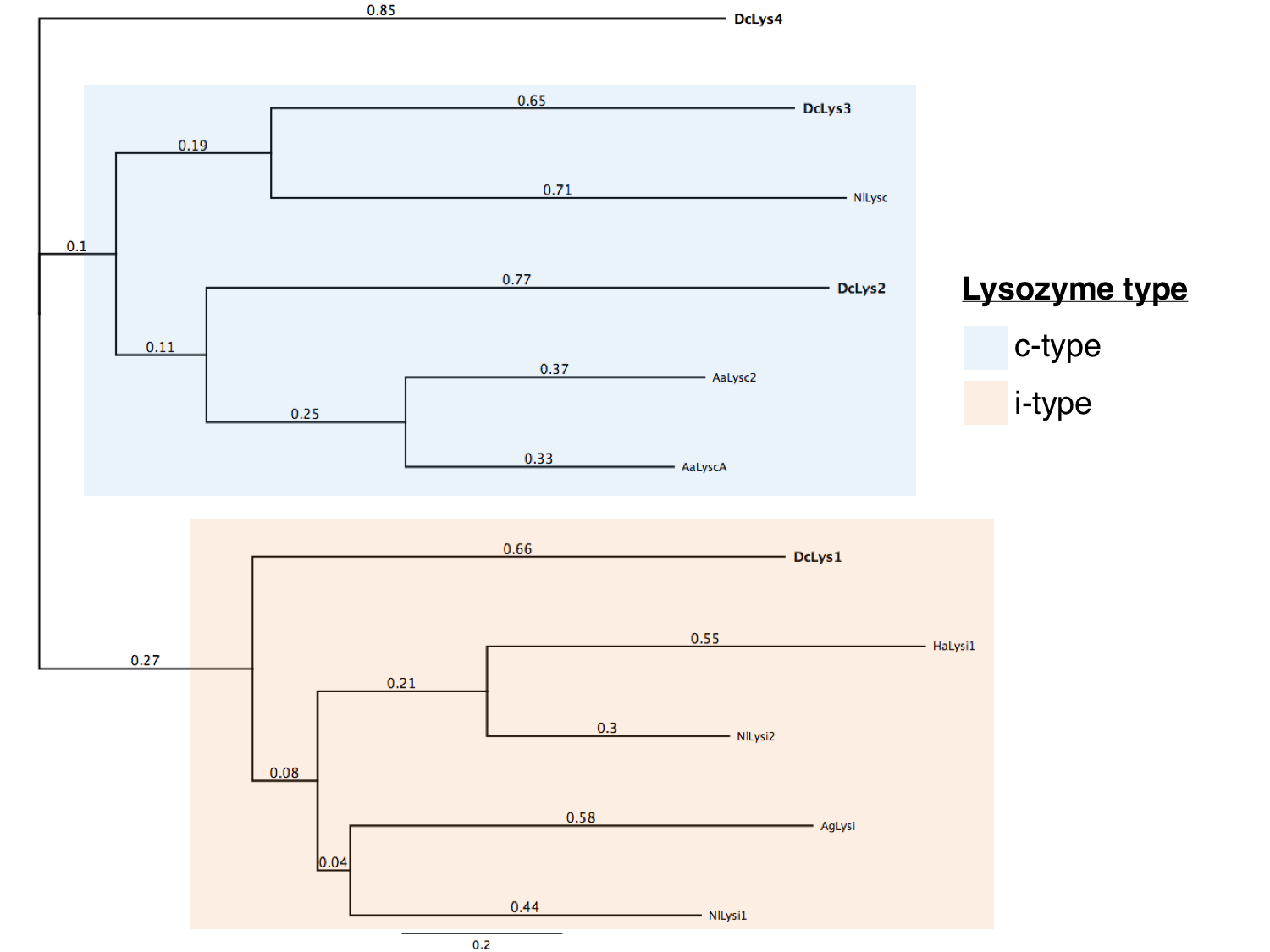


Figure S2. A phylogenetic comparison *D. citri* glutathione s-transferases with categorized GSTs from other insects. The phylogenetic tree was constructed using the Geneious (Version 7.1, Kearse et al., 2012) Tree Builder. Branch labels signify substitutions per site. Sequences used are listed in Table S5. Dm, *Drosophila melanogaster*; Ap, *Acyrthosiphon pisum*; Nl, *Nilaparvata* *lugens*; Bm, *Bombyx mori*; Dc, *Diaphorina citri*; Tm, *Tenebrio molitor*.


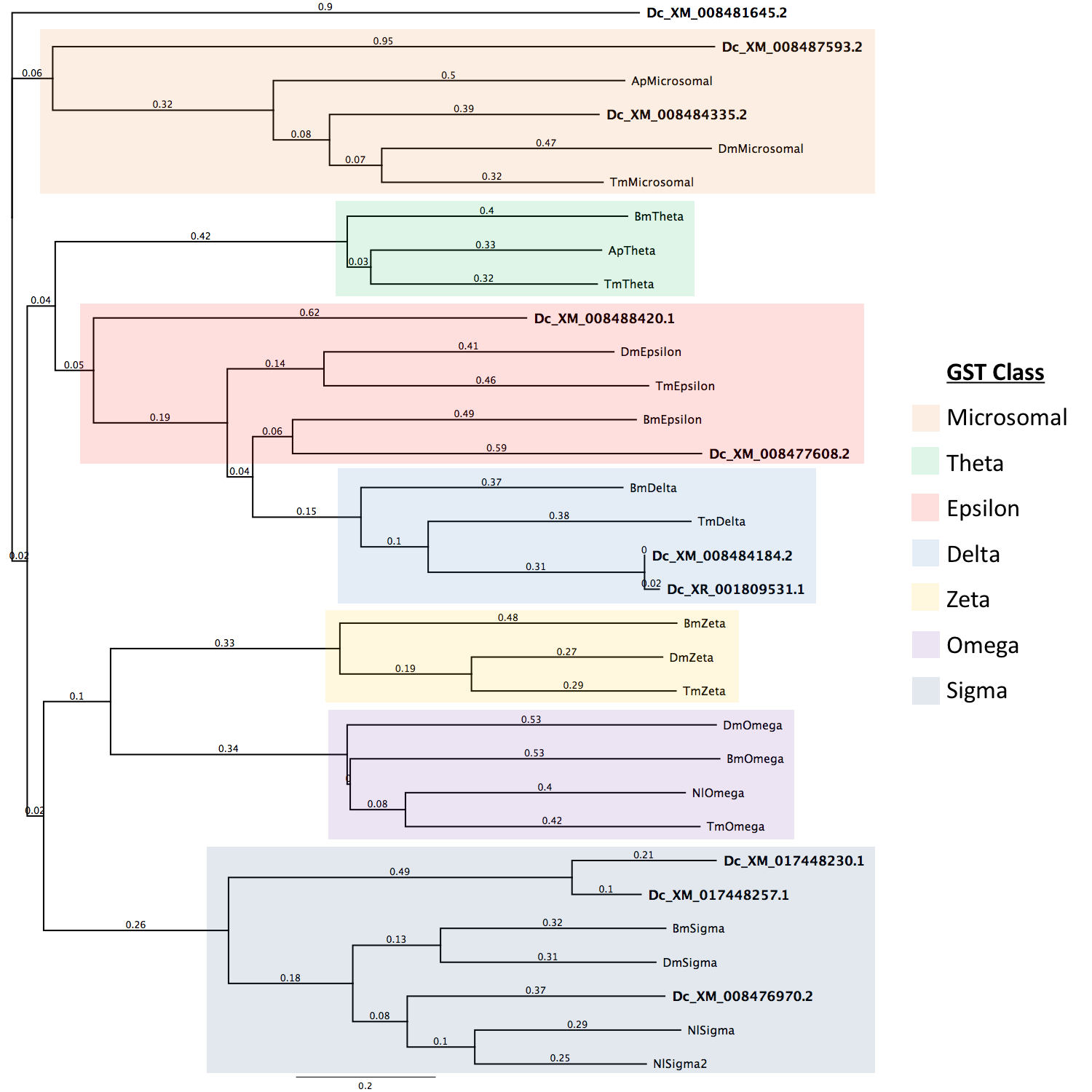


Table S1. Leucine rich repeat containing protein genes.

| Gene Prediction | Gene ID | Accessoin | Scaffold | Exon | Orientation | Best Match | Accession | Coverage | E-value | Identity |
| --- | --- | --- | --- | --- | --- | --- | --- | --- | --- | --- |
| Leucine-rich repeat neuronal protein 3-like | LOC103524588 | XM_008489614.1 | NW_007377544.1 | 2 | - | *Papilio machaon* | XP_014362368.1 | 64% | 9.00E-158 | 37% |
| Leucine-rich repeat and calponin homology domain-containing protein 1 | LOC103511613 | XM_008476341.2 | NW_007378164.1 | 12 | - | *Lasius niger* | KMR04571.1 | 10% | 6.00E-50 | 77% |
| Leucine-rich repeat neuronal protein 1-like | LOC103524447 | XM_008489463.1 | NW_007377536.1 | 1 | - | *Cimex lectularius* | XP_014246710.1 | 51% | 1.00E-35 | 33% |
| Leucine-rich repeat protein soc-2 homolog | LOC103514225 | XM_017446126.1 | NW_007378533.1 | 4 | - | *Neodiprion lecontei* | XP_015524096.1 | 58% | 3.00E-21 | 29% |
| Leucine-rich repeat protein soc-2 homolog | LOC103512680 | XM_008477458.2 | NW_007378305.1 | 1 | - | *Trichogramma pretiosum* | XP_014237336.1 | 74% | 0 | 72% |
| Leucine-rich repeat serine/threonine-protein kinase 1-like | LOC103516318 | XM_017445641.1 | NW_007377442.1 | 18 | - | *Melipona quadrifasciata* | KOX77506.1 | 82% | 0 | 49% |
| Leucine-rich repeat serine/threonine-protein kinase 1-like | LOC103521406 | XM_008486517.1 | NW_007382561.1 | 14 | + | *Tribolium castaneum* | XP_015840647.1 | 94% | 3.00E-108 | 53% |
| Leucine-rich repeat-containing protein 15-like | LOC103505977 | XM_008470362.1 | NW_007377449.1 | 9 | - | *Diuraphis noxia* | XP_015368265.1 | 79% | 1.00E-143 | 55% |
| Leucine-rich repeat-containing protein 15-like | LOC103524449 | XM_008489465.2 | NW_007377536.1 | 1 | + | *Riptortus pedestris* | BAN21113.1 | 94% | 2.00E-73 | 42% |
| Leucine-rich repeat-containing protein 4 | LOC103515182 | XM_008480113.2 | NW_007377484.1 | 6 | - | *Halyomorpha halys* | XP_014278698.1 | 77% | 4.00E-125 | 40% |
| Leucine-rich repeat-containing protein 40-like | LOC103506610 | XM_008471006.2 | NW_007377687.1 | 2 | + | *Orussus abietinus* | XP_012286484.1 | 83% | 4.00E-40 | 34% |
| Leucine-rich repeat-containing protein 51-like | LOC103515395 | XM_008480337.1 | NW_007378759.1 | 1 | + | *Neodiprion lecontei* | XP_015519846.1 | 53% | 6.00E-34 | 40% |
| Leucine-rich repeat-containing protein 58 | LOC103506716 | XM_008469334.1 | NW_007377694.1 | 7 | - | *Orussus abietinus* | XP_012276050.1 | 92% | 0 | 52% |
| Leucine-rich repeat-containing protein 59 | LOC103506234 | XM_008470620.2 | NW_007377661.1 | 8 | - | *Zootermopsis nevadensis* | KDR21947.1 | 28% | 5.00E-40 | 56% |
| Leucine-rich repeat-containing protein 70-like | LOC103520190 | XM_008485282.1 | NW_007380719.1 | 2 | - | *Diuraphis noxia* | XP_015371373.1 | 91% | 5.00E-79 | 57% |
| Leucine-rich repeat-containing protein let-4-like | LOC103522998 | XM_008488077.1 | NW_007391068.1 | 1 | - | *Acyrthosiphon pisum* | XP_001947265.4 | 99% | 3.00E-41 | 52% |
| Leucine-rich repeat, immunoglobulin-like domain and transmembrane domain-containing protein 2 | LOC103508847 | XM_008473426.2 | NW_007377874.1 | 1 | + | *Cimex lectularius* | XP_014259547.1 | 78% | 0 | 56% |
| Leucine-rich repeats and immunoglobulin-like domains protein 1 | LOC103522720 | XM_008487814.1 | NW_007388011.1 | 5 | - | *Pediculus humanus corporis* | XP_002422869.1 | 54% | 3.00E-90 | 49% |
| Leucine-rich repeats and immunoglobulin-like domains protein 1 | LOC103519591 | XM_017448246.1 | NW_007380241.1 | 5 | - | *Cephus cinctus* | XP_015602330.1 | 74% | 9.00E-107 | 72% |
| Immunoglobulin superfamily containing leucine-rich repeat protein-like | LOC103522224 | XM_008487326.1 | NW_007384838.1 | 1 | - | *Cimex lectularius* | XP_014259547.1 | 96% | 9.00E-104 | 36% |
| Chondroadherin-like protein | LOC103511885 | XM_008476628.2 | NW_007378195.1 | 3 | - | *Acyrthosiphon pisum* | XP_003240168.1 | 57% | 2.00E-67 | 75% |
| Chondroadherin-like protein | LOC103509718 | XM_017444285.1 | NW_007377956.1 | 12 | - | *Zootermopsis nevadensis* | KDR13390.1 | 73% | 0 | 63% |
| Plant intracellular Ras-group-related LRR protein 7-like | LOC103523687 | XM_008488708.1 | NW_007410309.1 | 1 | + | *Zootermopsis nevadensis* | KDR24519.1 | 98% | 4.00E-28 | 39% |
| leucine-rich repeat-containing protein 15-like | LOC103519705 | XM_008484797.2 | NW_007377504.1 | 3 | - | *Acyrthosiphon pisum* | XP_001950955.1 | 68% | 0 | 64% |
| leucine-rich repeat transmembrane neuronal protein 2-like | LOC103519820 | XM_008484910.2 | NW_007377504.1 | 1 | + | *Halyomorpha halys* | XP_014281332.1 | 75% | 4.00E-81 | 53% |
| leucine-rich repeat and fibronectin type-III domain-containing protein 5-like | LOC103524594 | XM_008489620.1 | NW_007377544.1 | 2 | - | *Papilio machaon* | XP_014362368.1 | 97% | 6.00E-77 | 36% |
| leucine-rich repeat-containing protein 4 | LOC103515182 | XM_008480113.2 | NW_007377484.1 | 6 | - | *Halyomorpha halys* | XP_014278698.1 | 77% | 4.00E-125 | 40% |
| leucine-rich repeat-containing protein 4-like | LOC103523176 | XM_008488244.1 | NW_007394029.1 | 1 | + | *Cimex lectularius* | XP_014249603.1 | 100% | 9.00E-81 | 61% |
| leucine-rich repeat-containing protein 57-like | LOC103518112 | XM_008483168.2 | NW_007379504.1 | 2 | + | *Orussus abietinus* | XP_012282226.1 | 45% | 1.00E-75 | 54% |
| leucine-rich repeat-containing protein 70-like | LOC103524129 | XM_008489157.1 | NW_007377444.1 | 4 | - | *Pediculus humanus corporis* | XP_002426747.1 | 96% | 6.00E-32 | 48% |
| immunoglobulin superfamily containing leucine-richrepeat protein-like | LOC103519828 | XM_008484919.1 | NW_007377504.1 | 2 | + | *Halyomorpha halys* | XP_014281332.1 | 72% | 1.00E-35 | 40% |
| leucine-rich repeat-containing protein 23-like | LOC103508301 | XM_008472844.1 | NW_007377820.1 | 5 | - | *Cimex lectularius* | XP_014240075.1 | 59% | 2.00E-11 | 44% |
| leucine-rich repeat-containing protein 71-like | LOC103505403 | XM_008469734.1 | NW_007377608.1 | 7 | - | *Amyelois transitella* | XP_013189037.1 | 24% | 0.005 | 29% |
| leucine-rich repeat-containing protein 15-like | LOC108254426 | XM_017449418.1 | NW_007468644.1 | 1 | - | *Halyomorpha halys* | XP_014283390.1 | 100% | 1.00E-42 | 72% |
| leucine-rich repeat-containing protein 3C-like | LOC108254411 | XM_017449403.1 | NW_007455415.1 | 1 | - | *Zootermopsis nevadensis* | KDR08672.1 | 98% | 5.00E-35 | 66% |
| leucine-rich repeat-containing protein DDB_G0290503 | LOC103521974 | XM_008487078.2 | NW_007383797.1 | 2 | - | none |  |  |  |  |
| leucine-rich repeat extensin-like protein 3 | LOC108253921 | XM_017448747.1 | NW_007382143.1 | 1 | - | none |  |  |  |  |
| MAP kinase phosphatase with leucine-rich repeats protein 1-like | LOC108253619 | XM_017447938.1 | NW_007379783.1 | 1 | - | *Nasonia vitripennis* | XP_008205334.1 | 23% | 5.00E-23 | 38% |
| leucine-rich repeat and immunoglobulin-like domain-containing nogo receptor-interacting protein 1-B | LOC103518517 | XM_017447832 | NW_007379650.1 | 3 | - | *Cimex lectularius* | XP_014241185.1 | 60% | 6.00E-21 | 47% |
| proline-, glutamic acid- and leucine-rich protein 1-like | LOC103523156 | XM_017449173.1 | NW_007393595.1 | 2 | + | *Zootermopsis nevadensis* | KDR10915.1 | 50% | 3.00E-41 | 29% |
| pollen-specific leucine-rich repeat extensin-like protein 1 | LOC103516526 | XM_017447091.1 | NW_007379016.1 | 5 | - | *Cimex lectularius* | XP_014261879.1 | 10% | 2.00E-06 | 61% |
| leucine-rich repeat-containing G-protein coupled receptor 6-like | LOC108253308 | XM_017447036.1 | NW_007378990.1 | 14 | - | *Cimex lectularius* | XP_014245069.1 | 96% | 2.00E-128 | 48% |
| proline-, glutamic acid- and leucine-rich protein 1-like | LOC108253110 | XM_017446471.1 | NW_007378690.1 | 5 | - | *Musca domestica* | XP_005177408.1 | 57% | 7.00E-04 | 28% |
| leucine-rich repeat-containing protein C10orf11 homolog | LOC103514446 | XM_017446224.1 | NW_007378574.1 | 4 | + | *Halyomorpha halys* | XP_014277759.1 | 71% | 6.00E-33 | 50% |
| leucine-rich repeat extensin-like protein 2 | LOC103514022 | XM_008478887.2 | NW_007378499.1 | 2 | - | *Halyomorpha halys* | XP_014278443.1 | 16% | 7.00E-07 | 44% |
| pollen-specific leucine-rich repeat extensin-like protein 1 | LOC103516199 | XM_008489532.2 | NW_007377539.1 | 6 | - | *Pediculus humanus corporis* | XP_002428765.1 | 9% | 1.1 | 42% |

Table S2. Free radical defense associated genes.

| Gene Prediction | Gene ID | Accession | Scaffold | Exon | Orientation | Best Match | Accession | Coverage | E-value | Identity |
| --- | --- | --- | --- | --- | --- | --- | --- | --- | --- | --- |
| Superoxide dismutase [Cu-Zn]-like | LOC103524169 | XM_008489173.1 | NW_007510402.1 | 1 | + | *Riptortus pedestris* | BAN20446.1 | 97% | 3.00E-27 | 84% |
| Superoxide dismutase [Cu-Zn]-like | LOC103507212 | XM_008471663.2 | NW_007377732.1 | 7 | - | *Diuraphis noxia* | XP_015368979.1 | 24% | 3.00E-50 | 59% |
| Superoxide dismutase [Cu-Zn]-like | LOC103509717 | XM_008474346.2 | NW_007377462.1 | 3 | - | *Coptotermes formosanus* | AGM32998.1 | 50% | 2.00E-78 | 79% |
| Superoxide dismutase [Cu-Zn]-like | LOC103516814 | XM_008481803.2 | NW_007379097.1 | 3 | - | *Acyrthosiphon pisum* | NP_001156243.1 | 51% | 5.00E-21 | 58% |
| Superoxide dismutase [Mn] 1, mitochondrial | LOC103506397 | XM_008470779.2 | NW_007377670.1 | 6 | + | *Bemisia tabaci* | AFW97646.1 | 59% | 3.00E-97 | 72% |
| Glutathione S-transferase-like | LOC103519619 | XM_017448257.1 | NW_007380255.1 | 8 | - | *Zootermopsis nevadensis* | KDR24333.1 | 68% | 2.00E-56 | 43% |
| Glutathione S-transferase-like | LOC103512218 | XM_008476970.2 | NW_007378243.1 | 5 | - | *Blattella germanica* | AEV23881.1 | 54% | 3.00E-98 | 66% |
| Glutathione S-transferase 1-like | LOC103519104 | XM_008484184.2 | NW_007379958.1 | 3 | - | *Riptortus pedestris* | BAN21522.1 | 56% | 4.00E-33 | 80% |
| Glutathione S-transferase 1-like | LOC103512823 | XM_008477608.2 | NW_007378321.1 | 3 | - | *Fopius arisanus* | XP_011314548.1 | 64% | 4.00E-40 | 47% |
| Glutathione S-transferase 1-like | LOC103510182 | XR_001809531.1 | NW_007378003.1 | 8 | + | *Nephotettix cincticeps* | AGV08610.1 | 60% | 4.00E-103 | 65% |
| Microsomal glutathione S-transferase 1-like | LOC103519249 | XM_008484335.2 | NW_007380041.1 | 3 | + | *Sogatella furcifera* | AFJ75813.1 | 63% | 6.00E-31 | 54% |
| Glutathione S-transferase 2-like | LOC103519526 | XM_017448230.1 | NW_007380189.1 | 7 | - | *Microplitis demolitor* | XP_014299347.1 | 81% | 2.00E-33 | 28% |
| Glutathione S-transferase D5-like | LOC103522490 | XM_008487593.2 | NW_007386240.1 | 2 | + | *Cimex lectularius* | XP_014249956.1 | 37% | 4.00E-09 | 30% |
| Glutathione S-transferase D7-like | LOC103523366 | XM_008488420.1 | NW_007398150.1 | 2 | + | *Papilio xuthus* | BAM18511.1 | 61% | 1.00E-19 | 57% |
| Glutathione S-transferase C-terminal domain-containing protein | LOC103516660 | XM_008481645.2 | NW_007377490.1 | 9 | + | *Polistes canadensis* | XP_014608075.1 | 85% | 1.00E-101 | 37% |
| Peroxidase-like | LOC103512366 | XM_017445382.1 | NW_007378262.1 | 21 | - | *Apis florea* | XP_012343058.1 | 67% | 1.00E-99 | 32% |
| Peroxidase-like | LOC103506469 | XM_008470857.2 | NW_007377674.1 | 6 | + | *Diuraphis noxia* | XP_015367109.1 | 71% | 2.00E-46 | 39% |
| Peroxidase-like | LOC103519578 | XM_017448240.1 | NW_007380232.1 | 9 | + | *Diuraphis noxia* | XP_015367109.1 | 78% | 2.00E-123 | 40% |
| Chorion peroxidase-like | LOC103512707 | XM_008477485.1 | NW_007378307.1 | 16 | + | *Diuraphis noxia* | XP_015367109.1 | 86% | 5.00E-85 | 34% |
| Phospholipid hydroperoxide glutathione peroxidase, nuclear-like | LOC103509202 | XM_008473799.1 | NW_007377909.1 | 5 | + | *Cimex lectularius* | XP_014250288.1 | 26% | 3.00E-34 | 47% |
| Glutathione peroxidase-like | LOC103520462 | XM_017448528.1 | NW_007381054.1 | 2 | + | *Zootermopsis nevadensis* | KDR10349.1 | 87% | 1.00E-25 | 61% |
| Phospholipid hydroperoxide glutathioneperoxidase | LOC103505868 | XM_017442611.1 | NW_007377638.1 | 4 | + | *Diuraphis noxia* | XP_015370806.1 | 74% | 2.00E-91 | 79% |

Table S3. Sequences used to create the phylogenetic tree in Figure 2.

| Name | Species | Gene | Accession |
| --- | --- | --- | --- |
| AgAttacin | *A. gambiae* | Attacin | XM_315630.4 |
| AgCec | *A. gambiae* | Cecropin | XM_311223.2 |
| AgDef1 | *A. gambiae* | Defensin 1 | XM_309352.4 |
| AgDef2 | *A. gambiae* | Defensin 2 | XM_556059.3 |
| ApLys | *A. pisum* | Lysozyme | XM_001949283.3 |
| BmIrp2 | *B. mori* | Immune response protein 2 | NM_001257010.1 |
| BmIrp1 | *B. mori* | Immune response protein 1 | NM_001098349.1 |
| BmLys | *B. mori* | Lysozyme | L37416.1 |
| DcHdd11 | *D. citri* | Hdd11 | XM_008473396.1 |
| DmAndropin | *D. melanogaster* | Andropin | NM_079848.2 |
| DmAtt2 | *D. melanogaster* | Attacin 2 | NM_079021.3 |
| DmAttacin | *D. melanogaster* | Attacin | NM_079005.3 |
| DmCec3 | *D. melanogaster* | Cecropin 3 | NM_079851.2 |
| DmCecropin | *D. melanogaster* | Cecropin | NM_079849.3 |
| DmDef2 | *D. melanogaster* | Defensin 2 | NM_078948.3 |
| DmDptB | *D. melanogaster* | Diptericin B | NM_079063.3 |
| DmDro3 | *D. melanogaster* | Drosocin 3 | NM_168020.2 |
| DmDrosocin | *D. melanogaster* | Drosocin | NM_079020.3 |
| DmLysB | *D. melanogaster* | Lysozyme B | NM_079158.2 |
| DmLysC | *D. melanogaster* | Lysozyme C | NM_080130.3 |
| DMLysS | *D. melanogaster* | Lysozyme S | NM_057481.4 |
| DmMetchnikowin | *D. melanogaster* | Metchnikowin | NM_079028.2 |
| NlDefA | *N. lugens* | Defensin A | KC355195.1 |
| NlDefB | *N. lugens* | Defensin B | KC355196.1 |
| NlLys | *N. lugens* | Lysozyme | KC355194.1 |
| NlLys1 | *N. lugens* | Lysozyme 1 | KC355196.1 |
| NlLys7 | *N. lugens* | Lysozyme 7 | KC355210.1 |
| NlReeler | *N. lugens* | Reeler | KC355218.1 |
| RpDefA | *R. prolixus* | Defensin A | AY196130.1 |
| RpDefC | *R. prolixus* | Defensin C | AY196132.1 |
| RpLys | *R. prolixus* | Lysozyme | EU250275.1 |

Table S4. List of sequences used in Figure S1.

| Name | Species | Lysozyme type | Accession |
| --- | --- | --- | --- |
| AaLysc2 | *A. aegyptii* | c-type | XM_001654183.2 |
| AaLyscA | *A. aegyptii* | c-type | XM_001664000.2 |
| AgLysi | *A. gambiae* | i-type | AY659931.2 |
| DcLys1 | *D. citri* | unknown | XM_017446560.1 |
| DcLys2 | *D. citri* | unknown | XM_017444286.1 |
| DcLys3 | *D. citri* | unknown | XM_008480591.1 |
| DcLys4 | *D. citri* | unknown | XM_008475598.1 |
| HaLysi1 | *H. axyridis* | i-type | KT380886.1 |
| NlLysi1 | *N. lugens* | i-type | KC355204.1 |
| NlLysi2 | *N. lugens* | i-type | KC355205.1 |
| NlLysc | *N. lugens* | Lysozyc-type | KC355194.1 |

Table S5. List of sequences used in Figure S2.

| Name | Species | GST Group | Accession |  |  |  |  |
| --- | --- | --- | --- | --- | --- | --- | --- |
| ApMicrosomal | *A. pisum* | Microsomal | NM_001162701.1 |  |  |  |  |
| ApTheta | *A. pisum* | Thet | NM_001162817.2 |  |  |  |  |
| BmDelta | *B. mori* | Delta | NM_001043718.1 |  |  |  |  |
| BmEpsilon | *B. mori* | Epsilon | NM_001043955.1 |  |  |  |  |
| BmOmega | *B. mori* | Omega | NM_001043941.1 |  |  |  |  |
| BmSigma | *B. mori* | Sigma | NM_001043529.1 |  |  |  |  |
| BmTheta | *B. mori* | Theta | NM_001114991.1 |  |  |  |  |
| BmZeta | *B. mori* | Zeta | NM_001046988.1 |  |  |  |  |
| Dc_XM_008476970.2 | *D. citri* | Unknown | XM_008476970.2 |  |  |  |  |
| Dc_XM_008477608.2 | *D. citri* | Unknown | XM_008477608.2 |  |  |  |  |
| Dc_XM_008481645.2 | *D. citri* | Unknown | XM_008481645.2 |  |  |  |  |
| Dc_XM_008484184.2 | *D. citri* | Unknown | XM_008484184.2 |  |  |  |  |
| Dc_XM_008484335.2 | *D. citri* | Unknown | XM_008484335.2 |  |  |  |  |
| Dc_XM_008487593.2 | *D. citri* | Unknown | XM_008487593.2 |  |  |  |  |
| Dc_XM_008488420.1 | *D. citri* | Unknown | XM_008488420.1 |  |  |  |  |
| Dc_XM_017448230.1 | *D. citri* | Unknown | XM_017448230.1 |  |  |  |  |
| Dc_XM_017448257.1 | *D. citri* | Unknown | XM_017448257.1 |  |  |  |  |
| Dc_XR_001809531.1 | *D. citri* | Unknown | XR_001809531.1 |  |  |  |  |
| DmEpsilon | *D. melanogaster* | Epsilon | NM_137479.3 |  |  |  |  |
| DmMicrosomal | *D. melanogaster* | Microsomal | NM_079957.4 |  |  |  |  |
| DmOmega | *D. melanogaster* | Omega | NM_139977.3 |  |  |  |  |
| DmSigma | *D. melanogaster* | Sigma | NM_001274111.1 |  |  |  |  |
| DmZeta | *D. melanogaster* | Zeta | NM_141637.3 |  |  |  |  |
| NlOmega | *N. lugens* | Omega | JQ917471.1 |  |  |  |  |
| NlSigma | *N. lugens* | Sigma | JQ917471.1 |  |  |  |  |
| NlSigma2 | *N. lugens* | Sigma | JQ917474.1 |  |  |  |  |
| TmDelta | *T. molitor* | Delta | KJ868731.1 |  |  |  |  |
| TmEpsilon | *T. molitor* | Epsilon | KJ868732.1 |  |  |  |  |
| TmMicrosomal | *T. molitor* | Microsomal | KJ868753.1 |  |  |  |  |
| TmOmega | *T. molitor* | Omega | KJ868745.1 |  |  |  |  |
| TmTheta | *T. molitor* | Theta | KJ868751.1 |  |  |  |  |
| TmZeta | *T. molitor* | Zeta | KJ868752.1 |  |  |  |  |
